# Intelligent Agent for Resource Allocation from Mobile Infrastructure to Vehicles in Dynamic Environments Scalable on Demand

**DOI:** 10.3390/s26020508

**Published:** 2026-01-12

**Authors:** Renato Cumbal, Berenice Arguero, Germán V. Arévalo, Roberto Hincapié, Christian Tipantuña

**Affiliations:** 1 Carrera de Telecomunicaciones, Universidad Politécnica Salesiana, Quito 170525, Ecuador; jcumbal@ups.edu.ec (R.C.); garevalo@ups.edu.ec (G.V.A.); 2 Carrera de Telecomunicaciones, Universidad Bolivariana, Medellín 050031, Colombia; roberto.hincapie@upb.edu.co; 3 Department of Electronics, Telecommunications, and Information Networks, Escuela Politécnica Nacional, Quito 170525, Ecuador; christian.tipantuna@epn.edu.ec

**Keywords:** AI, dynamic coverage, Q-learning, resource allocation, smart generic network controller, VANET, V2I

## Abstract

This work addresses the increasing complexity of urban mobility by proposing an intelligent optimization and resource-allocation framework for Vehicle-to-Infrastructure (V2I) communications. The model integrates a macroscopic mobility analysis, an Integer Linear Programming (ILP) formulation for optimal Road-Side Unit (RSU) placement, and a Smart Generic Network Controller (SGNC) based on Q-learning for dynamic radio-resource allocation. Simulation results in a realistic georeferenced urban scenario with 380 candidate sites show that the ILP model activates only 2.9% of RSUs while guaranteeing more than 90% vehicular coverage. The reinforcement-learning-based SGNC achieves stable allocation behavior, successfully managing 10 antennas and 120 total resources, and maintaining efficient operation when the system exceeds 70% capacity by reallocating resources dynamically through the λ-based alert mechanism. Compared with static allocation, the proposed method improves resource efficiency and coverage consistency under varying traffic demand, demonstrating its potential for scalable V2I deployment in next-generation intelligent transportation systems.

## 1. Introduction

Intelligent transportation systems (ITSs) have become essential for addressing the rapid growth of vehicular mobility worldwide. More than one billion vehicles currently circulate globally, and this number is expected to increase substantially in emerging regions such as China and Latin America [1,2]. In particular, vehicle ownership is projected to reach approximately 250 million in China, while Latin America is expected to grow to around 200 million vehicles by 2050 [3]. This sustained increase poses major challenges in traffic congestion, road safety, and environmental sustainability, and motivates the integration of advanced ITS technologies for more adaptive and efficient traffic management through connected and intelligent vehicles [2].

Modern intelligent vehicles operate within highly dynamic communication networks that generate and consume large volumes of data in real time. These data can be exploited using machine learning techniques to enable proactive traffic control strategies such as congestion forecasting, dynamic route recommendations, and adaptive traffic signal control. However, effectively deploying and operating these solutions remains challenging due to limitations in network planning, particularly in radio resource allocation and infrastructure adaptation to time-varying urban mobility demands. This gap highlights the need for intelligent and scalable approaches that ensure reliable V2I communication while adapting to changing traffic conditions [4,5].

Motivated by this context, this paper proposes an integrated framework for VANET infrastructure planning and dynamic radio resource management in georeferenced urban environments. First, macroscopic mobility modeling is applied to simulated scenarios to derive spatiotemporal vehicular demand and congestion patterns. Then, an ILP-based optimization model dynamically selects RSU deployment sites while considering coverage, capacity, and demand constraints. Finally, a Smart Generic Network Controller (SGNC) leverages reinforcement learning to adapt radio resource allocation over time, improving communication efficiency under fluctuating traffic loads.

**Figure 1:**
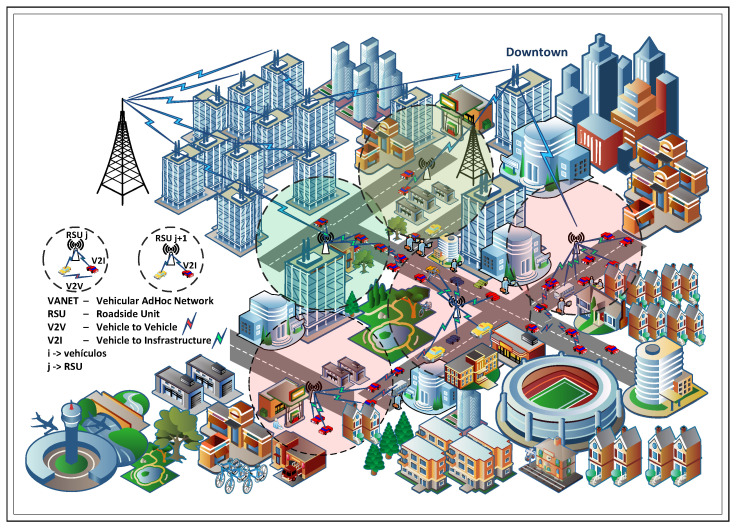
Example of a VANET architecture with different coverage radios.

The main contributions of this work are: (i) the development of a scalable and realistic VANET planning model that jointly considers coverage, capacity, and spatiotemporal demand derived from mobility patterns; and (ii) the design and implementation of a generic reinforcement-learning-based controller that dynamically allocates radio resources under infrastructure and channel constraints, supported by a comprehensive performance evaluation across diverse urban scenarios. These contributions advance the state of the art in vehicular communication systems and provide practical insights for deploying intelligent transportation networks in smart cities as explained in Figure 1.

The remainder of this paper is organized as follows. Section 2 discusses previous studies on this research approach. Section 3 formally presents the problem statement, optimization model variables, and the proposed SGNC. Section 4 presents the results obtained and their corresponding analysis. Finally, Section 5 presents the conclusions of this study, underlining the importance of the study’s findings.

## 2. Related Work

### 2.1. Mobility Modeling and Traffic Simulation

In vehicular networks, each vehicle can be considered a mobile node whose movement follows predefined road structures, making its trajectory partially predictable. Modern vehicles integrate computing, communication, sensing, and navigation systems developed collaboratively by industry, academia, and governmental institutions. These systems enable the creation of prototypes and standards that enhance communication performance, which is typically evaluated through simulation due to the high cost of real-world deployment [6,7].

Traffic simulators generate realistic geospatial environments—including roads, buildings, and natural features—and are essential for evaluating VANET performance at both macroscopic and microscopic levels. Tools such as OpenStreetMap (OSM) are widely used to define realistic planning areas for mobility and communication analysis. Mobility simulations can be classified into random models, flow-based models, behavioral models, and trace-based models [8,9] to determine the planning area for both the mobility and communication networks, thereby creating an ideal scenario for analysis. Mobility simulators are divided into several models: random, flow, traffic, behavioral, and trace.

In vehicular traffic, various macroscopic models have been studied, focusing on the global analysis of traffic to calculate parameters such as road capacity, vehicle distribution, and traffic density [10,11].Microscopic models, on the other hand, analyze the movement of each vehicle individually, considering its physical capabilities [12,13,14,15,16,17,18,19]. Although most studies have focused on microscopic aspects, this can lead to unrealistic models due to the complexity of these scenarios. By providing a global view of the system, macroscopic models are easier to manage and require fewer computational resources, unlike microscopic models, which are more detailed but require greater processing power [20].

Until now, researchers have primarily focused on microscopic aspects, overlooking macroscopic ones. There are several realistic mobility models based on simulated traces, real-world maps, and integration of existing models. Real-world traces are obtained using devices such as GPS, while artificial traces and traffic are modeled using software [7,20]. On the other hand, the simulator of urban mobility (SUMO), widely recognized and used in the literature, is a reliable and trusted tool. It uses the Krauss model to calculate future vehicle speeds, becoming a flow simulator based on a microscopic approach. Recently, Machine Learning (ML) methods have been incorporated to improve the realism of simulations, allowing for more accurate modeling of movement patterns in complex urban and road environments.

### 2.2. Congestion Analysis and ML-Based Traffic Prediction

Traffic congestion is a problem that affects both drivers and passengers, increasing travel times and generating economic, environmental, and health losses. Furthermore, it negatively impacts communication in vehicular networks, creating congestion at the data level. This phenomenon has been studied since 2010 [21], and more precise methods for detecting recurring and non-recurring congestion are being developed, allowing events to be monitored in real-time. In this sense, statistical inference and ML algorithms play a crucial role, as they allow for obtaining relevant parameters and applying proactive strategies to improve traffic conditions [22].

### 2.3. VANET Communication Models: V2V, V2I, and ITS Integration

VANETs, on the other hand, are equipped with computing modules and wireless devices that allow them to integrate into an ITS, meeting strict requirements for low latency and high transfer speeds [22]. Their role in expanding connected and autonomous vehicles is significant, V2V and V2I communications are transforming the transportation system and the road environment. V2V communications enable vehicles to communicate directly with each other or through multi-hop schemes, thereby reducing the need for numerous RSUs and enhancing coverage without compromising network performance. V2I communications connect vehicles with fixed units within the road infrastructure, i.e., with RSUs. Integrating V2V and V2I technologies with ITSs optimizes network efficiency and enhances safety, instilling confidence in the effectiveness of these technologies [23,24,25,26,27,28,29,30,31,32,33,34,35].

In scenarios with few RSUs, optimizing their distribution is not a priority. However, in larger areas, it is essential to carefully plan their deployment to maximize coverage without excessively increasing installation costs. This balance between coverage and cost requires strategic planning to ensure the network remains efficient and functional [36]. To optimize RSU placement, various solutions have been proposed, including techniques such as linear programming and multi-objective evolutionary algorithms. These methodologies aim to improve network coverage while minimizing the number of RSUs required, contributing to resource savings [37,38]. Spatiotemporal coverage strategies based on the representation of vehicle mobility patterns have also been proposed. In this scenario, optimal RSU locations are calculated to cover these patterns with the fewest possible units, improving efficiency compared to previous studies [39]. However, scalability remains a significant challenge, especially in large networks.

### 2.4. RSU Deployment and Infrastructure Optimization

The efficient design and deployment of VANET infrastructure ensures reliable communications in vehicular networks. The strategic placement of RSUs and resource optimization are crucial for improving coverage, reducing costs, and maximizing network performance in high-mobility environments. This involves variability in vehicle density depending on the location (urban or peripheral areas) and the time of day (peak or off-peak), and requires flexible and robust resource management schemes. These schemes must adapt to such variations and ensure the efficient use of available resources because vehicles function as network nodes and information sources [40]. VANET communication standards have been developed globally, such as DSRC in the US and ITS-G5 in Europe, based on IEEE 802.11p [20] technology. However, these standards are not without their challenges. Issues such as channel access delays, lack of quality of service, and short-lived connections between vehicles and infrastructure are significant hurdles that need to be overcome. To address these challenges and take advantage of the high penetration of cellular networks, the 3GPP project has started investigating the use of LTE for vehicle to everything (V2X) services, which offer a promising alternative with advantages over IEEE 802.11p, such as increased mobility, congestion resilience, higher multiplexing capacity, and better coverage [40]. Despite these advantages, technical challenges remain, especially in designing efficient resource allocation schemes that meet bandwidth, power consumption, and energy efficiency requirements. As V2I data traffic is expected to increase rapidly in the coming years, the need for efficient solutions to these problems becomes even more urgent. Most current studies approach the problem from a static perspective without considering dynamic changes in the radio channel, which limits the adaptive capacity of the algorithms [41].

### 2.5. Resource Allocation in Vehicular Networks

Recently, there has been a significant increase in studies proposing prediction-based protocols to improve the efficiency and reliability of vehicular networks. These advances, driven by artificial intelligence, ML, and particularly in deep learning, have achieved remarkable progress in image classification, video games, and robotics. These technologies enable the development of intelligent systems that operate in complex environments, analyzing large volumes of data to identify patterns relevant to vehicular network problems, thereby helping networks make more informed decisions [42]. However, adapting these tools to the particular characteristics of vehicular networks, such as their high mobility, remains a significant challenge and a promising avenue for future research.

Flexible and scalable resource allocation strategies are essential in vehicular networks to ensure service quality and meet user needs. However, many vehicular networks still rely on fixed allocations, failing to account for variations in traffic and coverage [43]. Vehicles’ high mobility leads to constant changes in network topology and the number of users within the range of RSUs, making efficient resource allocation a challenge. As the number of connected vehicles increases, the demand for spectrum exceeds availability, underscoring the critical need for improved resource allocation strategies [44]. Some researchers have developed optimization models to maximize user utility, considering traffic variability and energy efficiency. A key strategy in this research has been the use of distributed algorithms, which have significantly reduced computational complexity. These algorithms, implemented using techniques such as augmented Lagrangian, have proven effective in achieving near-optimal solutions in large-scale networks. Other studies have applied game theory to model resource allocation as a noncooperative congestion game, seeking fairness in spectrum distribution. Models based on semi-Markov decision processes and hierarchical schemes, as well as those based on the Nash bargaining game, have also been explored. Despite these advances, efficiently increasing system capacity remains challenging, especially in V2I communications. Dynamic resource allocation becomes even more complicated when past decisions influence future ones, because vehicular environments are highly variable and limited available resources. Therefore, heuristic and iterative methods have been proposed to maximize V2I communication capacity, considering interference and applying advanced optimization algorithms [45].

### 2.6. Reinforcement Learning for Dynamic Resource Management

The exponential growth of data traffic in the coming years demands more efficient solutions, such as dynamic spectrum access or the formation of vehicle clusters to share resources. These solutions have proven effective, although they still have limitations. Reinforcement learning (RL) has emerged as a key tool for optimizing resource allocation in vehicular networks. Its adaptability in handling dynamic environments, where complete information is not always available, makes it a promising solution [36,46,47,48]. This technology can reduce congestion and improve data delivery efficiency, giving us confidence in its potential [37,38,49,50].

The limited bandwidth of VANET communications, combined with high packet loss rates and short contact windows between vehicles, restricts the amount of data that can be transferred. This condition shows the need for more efficient spectrum management and real-time network monitoring to detect and correct inefficient bandwidth usage, thereby improving system performance [38]. In this sense, RL emerges as an innovative alternative to traditional mathematical models, allowing vehicular networks to adapt to rapid changes and optimize resource allocation. Unlike centralized approaches, which can minimize packet collisions, decentralized models are distinguished by their simplicity and fast learning. As artificial intelligence advances, its integration into vehicular networks promises to transform resource management, providing more reliable and efficient communications.

### 2.7. Summary and Motivation

Table 1 summarizes representative studies, highlighting their main limitations. Overall, the literature reveals that: (1) RSU deployment, mobility modeling, and resource allocation are often addressed separately; (2) dynamic variations in demand and channel conditions are rarely integrated into a unified framework; and (3) RL approaches lack scalability and real-world mobility realism.

This motivates the development of a scalable RL-based intelligent agent capable of adapting resource allocation dynamically under realistic mobility and infrastructure constraints.

## 3. Methodology

**Figure 2:**
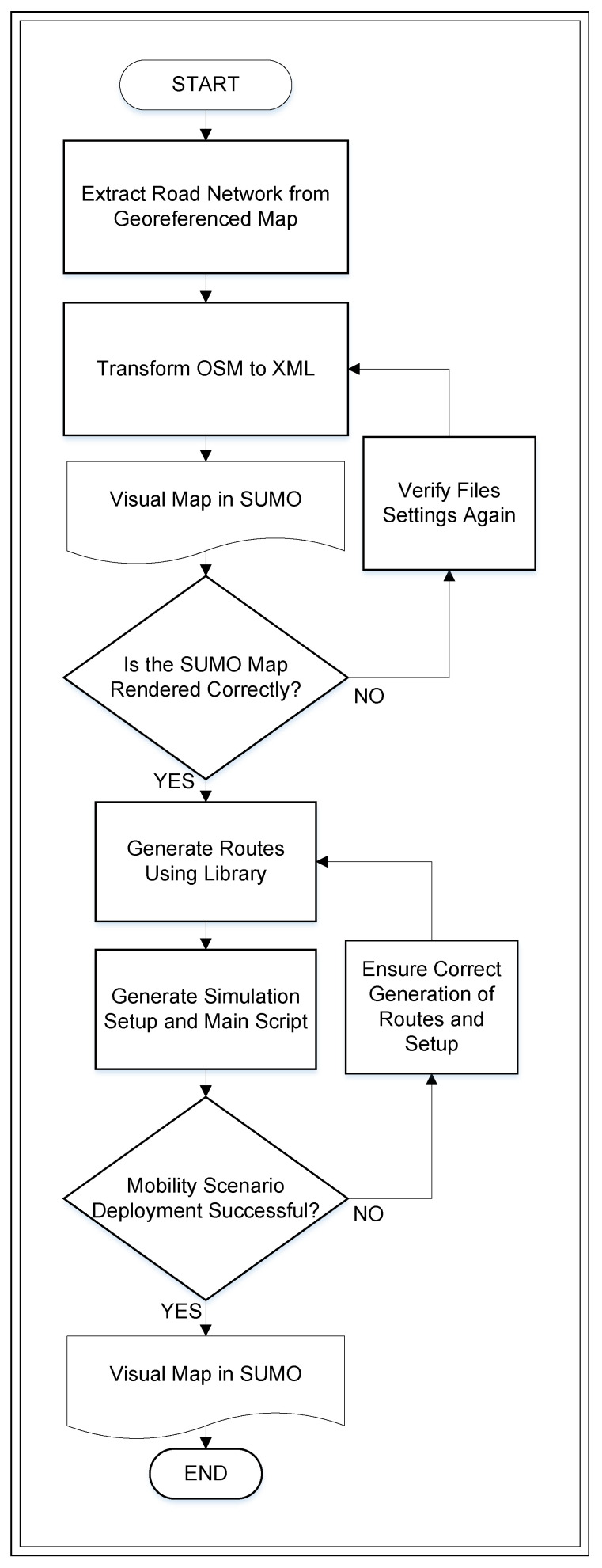
Flowchart of processes for modeling traffic in a vehicular network.

RSU planning requires a methodological model that combines traffic modeling and optimization strategies to ensure reliable coverage, efficient resource allocation, and service continuity in urban areas. Vehicular mobility introduces fluctuations in traffic density and connectivity, which complicate static planning. Furthermore, the growing demand for V2I services requires adaptive mechanisms for resource allocation and network optimization. To address this challenge, a hybrid simulation model has been created that combines microscopic and macroscopic traffic analysis, generating scenarios based on the existing road infrastructure and modeling vehicular flow according to road characteristics. Figure 2 illustrates the flowchart outlining the processes, from geospatial data conversion to simulation execution in SUMO.

The modeling process in Figure 2 follows several stages. First, geographic data containing structured road network information is obtained from OSM for a selected 1.9 km × 1.3 km area of a dense urban environment. This data is organized into a processable format and integrated into the SUMO traffic simulator. The simulation analyzes traffic patterns, including vehicle density, average speed, and intersection behavior. From this data, the demand for V2I infrastructure is estimated, and RSU placement is optimized, ensuring efficient deployment in urban environments with high mobility. The algorithm flow begins with the acquisition of geospatial data, followed by the generation of vehicle mobility using the *randomTrips.py* script, a key step in the process. Finally, the *sumocfg* configuration file integrates the components necessary to run and visualize the simulation.

In the initial simulation phase, random vehicle trajectories are generated from the converted map, taking into account key parameters such as the total simulation time and the edge weight probability, which is based on road length. The *sumocfg* configuration file is rebuilt, centralizing the simulation in SUMO and incorporating the previously generated road infrastructure (*net*) and vehicle route (*rou*) files. The *sumocfg* file specifies the start and end times of the simulation, ensuring its execution according to the defined parameters. Algorithm 1 below shows instructions for configuring the mobility generator, enabling the creation of dynamic scenarios in SUMO.
**Algorithm 1** Optimized ILP model for RSU-based coverage**Require:** Vehicle coordinates (x,y) and RSU coordinates $\left(x_{rsu},y_{rsu}\right)$ **Ensure:** Optimized ILP model for network coverage
  1:**Initialize parameters:**  2:R←200                       ▹ Coverage radius  3:N← length of *x*                     ▹ Number of vehicles  4:M← length of $x_{rsu}$                   ▹ Number of RSUs  5:Cap←12                         ▹ Capacity per RSU  6:**Step 1: Compute Euclidean distance**  7:**for** i←1 to *M* **do**  8:      **for** j←1 to *N* **do**  9:            Compute distance $d_{i,j}$ between vehicle *j* and RSU *i*10:      **end for**11:**end for**12:**Step 2: Define ILP model**13:Open file to store equations14:**Step 3: Objective function**15:Minimize total number of active RSUs:16:$\min\sum_{i=1}^{M}Z_{i}$17:**Step 4: Coverage constraints**18:Ensure at least *p* percentage of users are covered:19:$\sum_{j=1}^{N}Y_{j}\geq N\cdot p$20:**Step 5: Unique connection constraint**21:**for** j←1 to *N* **do**22:      temp←0                       ▹ Coverage flag23:      **for** i←1 to *M* **do**24:            **if** $d_{i,j}\leq R$ **then**25:               temp←126:               Print constraint equation including $Z_{i}$27:            **end if**28:      **end for**29:      **if** temp==0 **then**30:            Print equation forcing $Y_{j}=0$31:      **end if**32:**end for**33:**Step 6: Variable domains**34:**for** i←1 to *M* **do**35:      Print constraint: $Z_{i}\in \left\{0,1\right\}$               ▹ Binary variable36:**end for**37:**for** j←1 to *N* **do**38:      Print constraint: $Y_{j}\in \left\{0,1\right\}$39:**end for**40:Close file after saving equations


Once the dynamic vehicle mobility simulation has been developed, the vehicle trace from the generated files will be extracted. This trace contains essential information, such as the location and speed of each vehicle on the network at each instant in time, allowing the creation of realistic communication scenarios. The configuration file (*.sumocfg*) stores detailed records of vehicle movement, where parameters such as speed, frequency, accuracy, and data formats are adjusted. These settings allow the traces to be converted into formats compatible with other simulators, such as OMMET, NS2, and NS3, in the *.tlc* format, demonstrating the adaptability of our system. The extracted data is organized and filtered into position coordinates (x, y) to identify each vehicle and correlated times. This data is structured into columns within a temporary file *temp.dat*, as shown in Table 2. A vehicle counting module is incorporated for each analysis sector, utilizing the obtained traces. This module records the number of vehicles present at each instant in time $t_{i}$, storing the data in a vector. Each analysis sector has a defined coverage radius, delimiting a specific area within the planning map. This enables the visualization of vehicle distribution in different regions and estimates the number of vehicles in each area throughout the simulation.

### 3.1. Vehicle Infrastructure Optimization

We analyze the communications infrastructure for the coverage model to determine the minimum number of RSUs needed to cover vehicular demand. This analysis involves evaluating the number of vehicles in the planning area and their distribution across each antenna using the Set Cover algorithm with integer linear programming, based on the Greedy algorithm. To achieve this, we consider mobility and vehicular flow as given between a vehicle and an RSU within the V2I domain, aiming to identify the minimum set of RSUs that maximizes coverage while minimizing cost. The mobility simulation is run in time intervals $t_{i}$, allowing for dynamic and periodic coverage analysis. In addition, an ML model is implemented with a network controller that first learns the dynamics of vehicular traffic and subsequently optimizes radio resource allocation.

Deploying vehicular infrastructure in a VANET requires establishing a simulated road system to evaluate the optimal deployment of RSUs. This system considers fixed or variable coverage radio and limited capacity. The main objective is to maximize vehicle connectivity within the planning area, ensuring efficient coverage and adequate capacity for simultaneous communication between multiple nodes.

The optimization model is based on a set of *N* vehicles $Y_{j}$, which move dynamically along the road network during a time interval $t_{i}$. These vehicles determine traffic flow and congestion, directly influencing the structure of the mobility network and the demand for V2I communications. From their trajectories $Y_{j}=\left\{y_{1},y_{2},y_{3},\ldots ,y_{N}\right\},$ the positions $\left(x_{j},y_{j}\right)$ are recorded as a function of time $t_{i}$, where *i* is the index that represents a specific moment in a temporal sequence.

A set of candidate sites *Z* of size *M* is also defined, representing strategic locations within the planning area where RSUs can be installed. Their activation depends on traffic demand and the capacity of the infrastructure. Each candidate site *i* is associated with a coverage radius $R_{\mathrm{rsu}}$ and has fixed coordinates $\left(x_{{\mathrm{rsu}}_{j}},y_{{\mathrm{rsu}}_{j}}\right)$, where

$$Z=\left\{\left(x_{{\mathrm{rsu}}_{1}},y_{{\mathrm{rsu}}_{1}}\right),\left(x_{{\mathrm{rsu}}_{2}},y_{{\mathrm{rsu}}_{2}}\right),\ldots ,\left(x_{{\mathrm{rsu}}_{M}},y_{{\mathrm{rsu}}_{M}}\right)\right\}.$$

Throughout the optimization process, the optimal distribution of RSUs is determined to fully cover the planning scenario. The final selection of locations is based on a dynamic analysis, ensuring that the infrastructure is deployed efficiently and responds to the real needs of vehicular traffic. To minimize the number of RSUs, a minimum coverage of at least a percentage ρ is guaranteed during each time interval $t_{i}$.

For this purpose, the set of candidate sites is defined as

$$Z=\{z_{1},z_{2},z_{3},\ldots ,z_{M}\},$$

where each element $z_{i}$ (i=1,2,…,M) represents a candidate site with fixed coordinates $\left(x_{{\mathrm{rsu}}_{i}},y_{{\mathrm{rsu}}_{i}}\right)$. To activate an RSU, the binary variable $\alpha_{i,j}$ is used; *i.e.*, if $\alpha_{i,j}=1$, the RSU is active, otherwise it is inactive.

A minimum coverage percentage ρ is required in the planning scenario, and the variable $Z_{i}$ identifies the activated candidate sites. The goal is to minimize the number of active RSUs from the total number *M*. This is formulated in Equation (1), which defines the objective function for RSU activation.

(1) $$\begin{array}{cc} \min:\; & \sum_{i=1}^{M}Z_{i} \end{array}$$

The given objective function guarantees compliance with the coverage and efficiency requirements for RSU deployment. To achieve this, certain restrictions must be established. The first is defined in Equation (2), which establishes the minimum coverage condition. This restriction ensures that the V2I infrastructure always covers a minimum percentage of total vehicular demand at each instant in time $t_{i}$.

(2) $$\sum_{j=1}^{N}Y_{j}\geq N*\rho$$

where, *N* is the total number of vehicles in the planning area and ρ is the minimum percentage of coverage required. Ensuring that the number of vehicles covered at any given time is no less than ρ of the total demand indicates that the infrastructure is efficient, providing adequate coverage for system requirements.

The second constraint is given in Equation (3), where the connectivity condition between each vehicle $Y_{j}$ and the RSUs within the planning area is established, ensuring that each vehicle *j* is associated with a single RSU *i*, as long as it is within the coverage radius of that RSU.

(3) $$\sum_{i=1}^{M}X_{i,j}*\alpha_{i,j}\geq Y_{j}\;;\;\forall j\in Y$$

Equation (3) evaluates whether vehicle *j* is within the coverage area of RSU *i*, based on the Euclidean distance between them. For a connection to be considered valid, the distance must be less than the coverage radius of the RSU. In scenarios where a vehicle is located within the overlapping coverage areas of multiple RSUs, the selection of the optimal RSU is determined by the criterion of minimum distance.

The binary variable $\alpha_{i,j}$ plays a central role in this process. It takes the value 1 if vehicle *j* is within the coverage radius of RSU *i*, and 0 otherwise.

Once connectivity has been validated through Equation (3), Equation (4) enforces a constraint ensuring that each vehicle connects to only one RSU. This condition is critical to avoid simultaneous associations with multiple RSUs, which could result in communication interference or resource conflicts. Therefore, Equation (4) ensures a one-to-one assignment between vehicles and RSUs, promoting system stability and communication efficiency.

(4) $$\begin{array}{c} \sum_{i=1}^{M}X_{i,j}\leq 1;\;\forall j\;\in Z \end{array}$$

Likewise, Equation (5) limits the capacity of RSUs relative to demand. Specifically, this constraint ensures that no RSU exceeds its available resource capacity to meet vehicular demand at any given time point. This ensures that each RSU can efficiently manage vehicle connections without overloading the infrastructure.

(5) $$\begin{array}{c} \sum_{j=1}^{N}X_{i,j}\leq \mathrm{cap}\times Z_{i} \end{array}$$

The sum $X_{i,j}$ counts how many RSUs have an active connection to vehicle *j*, and the cap parameter represents the maximum number of connections that an RSU can support as s maximum capacity. Finally, the constraint in Equation (6) defines the domain of the variables $Y_{j}$ and $Z_{i}$ so that they are within the logical and operational limits of the system.

(6) $$\begin{array}{c} Z_{i}\in \left\{0,1\right\},\;Y_{j}\in \left\{0,1\right\},\;X_{i,j}\in \left\{0,1\right\} \end{array}$$

Once the objective function and its constraints have been defined, the optimal placement of RSUs must be determined using Algorithm 1, which is used for infrastructure deployment optimization. This algorithm minimizes the number of activated units while ensuring adequate coverage.

In Algorithm 1, optimal candidate sites are identified as a function of time, considering the stochastic nature of the model. This means that both the model and the algorithm must address a dynamic sequence of events, where parameters change over time due to vehicular mobility and traffic variability, to ensure that the selection of active candidate sites optimizes network performance at each instant. RSU locations and their resources must be dynamically adjusted based on traffic demand and vehicular mobility to achieve this. At each time step $t_{i}$, the algorithm must evaluate the current conditions, such as vehicle density, traffic flow, and congestion, to determine which candidate sites should be activated and which RSUs should provide coverage for vehicles.

### 3.2. Dynamic Allocation of Radio Resources in the Infrastructure

The Q-learning reinforcement learning algorithm is implemented for dynamic resource allocation, allowing the system to learn and improve through interaction with its environment. This environment consists of a planning area characterized by the dynamics of RSU antenna mobility and demand, which is determined by the number of vehicles and varies over time $t_{i}$. To optimize the use of available resources, a controller agent is introduced that manages resource allocation based on time and space. The agent makes decisions about resource allocation and rewards, reflecting the impact of each action on system performance. As the process progresses, the system adjusts its strategy to maximize resource allocation efficiency. The agent refines its approach through multiple iterations based on the rewards it obtains, ensuring continuous improvement in resource allocation. It continuously improves resource allocation, allowing the system to adapt to environmental changes and ensuring stable and efficient communication in vehicular networks, Table 3 and Table 4. Notation and key symbols defining the state, action, and reward components of the SGNC learning-based control framework.

**Figure 3:**
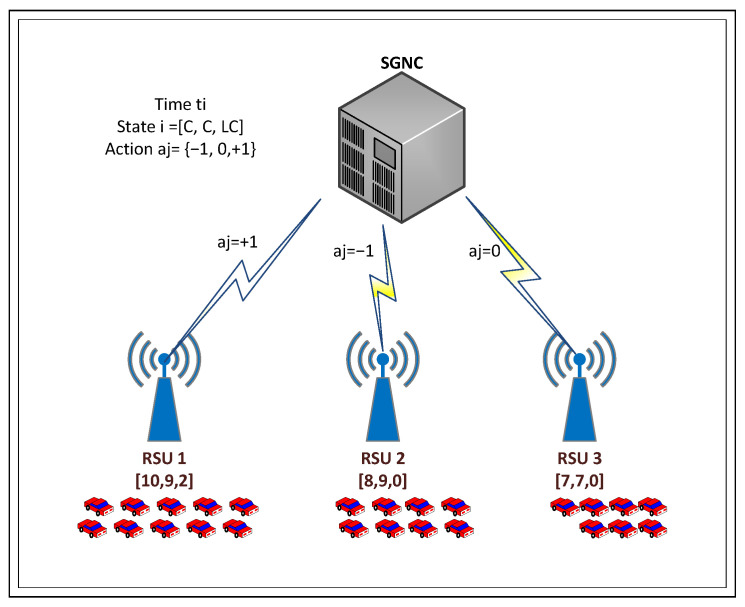
System state with SGNC actions in resource allocation.

The set of actions defines the decisions the system can take to modify its state; these are represented by the values applied to the current state *S* to reach a new one. The actions are defined as A∈{−1,0,1}, where −1 reduces the allocated resources, 0 keeps the resources unchanged, and 1 increases them. The states are divided into $S_{a}$, which represents the current state at time $t_{i}$, and $S_{n}$, which corresponds to the new state after applying an action $a_{j}$ at time $t_{i+1}$. The state domain is defined as

$$S=\{\left(0\text{-}C,\;0\text{-}C,\;0\text{-}LC\right)|C\in Z^{+}\},$$

where *C* is a positive integer corresponding to the demand components and allocated resources, which vary dynamically depending on vehicular mobility and the capacity of each antenna at time $t_{i}$. As shown in Figure 3, the demand (number of vehicles) for the antennas constitutes the system’s state vector. The controller, responsible for resource allocation, makes decisions to distribute resources among antennas, with the options of allocating one unit (+1), taking no action (0), or removing one unit (−1). When an action is executed, a reward $r_{i}$ is obtained. Table 3 describes the variables and arrays used in the context of dynamic resource allocation through the proposed SGNC agent. This notation supports the mathematical and algorithmic formulation of the learning process.

Algorithm 2 introduces the reinforcement learning approach that trains the SGNC agent, allowing it to improve its resource allocation strategy through interaction with the environment and feedback from rewards. The algorithm begins by loading the Q matrix, which is the result of the SGNC agent’s learning process. Subsequently, an output matrix with three columns is generated in the second phase. Specifically, the first column presents the cumulative rewards associated with Action 1 (i.e., action −1), which corresponds to the reduction of allocated resources. The second column displays the cumulative rewards resulting from Action 2 (i.e., action 0), in which the SGNC takes no action. The third column shows the cumulative rewards for Action 3 (i.e., action +1), which represents the allocation of additional resources to the RSUs. These values provide insight into the learned policy and are used during the third phase of the algorithm to generate the corresponding policy visualization.
**Algorithm 2** Reinforcement learning algorithm for SGNC agent training  1:**Initialize parameters:**  2:$N_{r}=10$, C=12, PORC=70  3:$CAP\leftarrow \lfloor \left(N_{r}\cdot C\right)\cdot \left(PORC/100\right)\rfloor$  4:$LC\leftarrow N_{r}\cdot C-CAP$  5:Generate possible states [C,C,LC]∈|C×C×LC|  6:**for all** states **do**  7:       Indest(states)=i  8:**end for**  9:Load MDP-based transition matrix10:Define action space actions={−1,0,1}11:Initialize Q-table and visit counters12:Set hyperparameters: num= 200,000, γ=0.7, α=0.7, β=0.5, $\beta_{n}=0.01$13:Compute decay factor: $K={\left(\beta /\beta_{n}\right)}^{\left(1/num\right)}$14:Initialize: asig=0, Λ=015:**Main loop: Simulation execution**16:**for** t=1 to num **do**17:       β=β/K18:       Generate random demand: state←rand([0,C])19:       Initialize assignment: asig←rand([0,C])20:       Enforce capacity limits21:       Λ←min(max(∑asig−LC,0),LC)22:       episodes=10023:       **for** e=1 to episodes **do**24:          **for all** *i* in RSU set **do**25:             Sample next state and compute state–action index26:             **Epsilon-greedy action selection:**27:             **if** rand()<β **then**28:                  Select random action29:             **else**30:                  Select best action: [vmax,imax]=max(Q(iSa,:))31:             **end if**32:             Update assignment: asig(i)←asig(i)+a33:             penalty←034:             **if** asig(i)<0 **then**35:                  asig(i)←0, penalty=−1036:             **end if**37:             **if** asig(i)>C **then**38:                  asig(i)←C, penalty=−1039:             **end if**40:             Update load imbalance:41:             Λ←min(max(∑asig−LC,0),LC)42:             Define next state: Sn=[state(i),asig(i),Λ]43:             Update next state index and visit count44:             Compute reward Rwrd according to Equation (7)45:             **if** Rwrd==0 **then**46:                  Rwrd←1047:             **end if**48:             Update Q-learning rule:49:             Q(iSa,a)←Q(iSa,a)+α(Rwrd+γmax(Q(iSn,:))−Q(iSa,a))50:          **end for**51:       **end for**52:       Save simulation results53:       Print current time step54:**end for**

The Q-Learning algorithm is a model-free algorithm that learns from the optimal policy that is built during training. This is an efficient method that does not require prior knowledge of the environment. The Bellman equation facilitates the implementation of the algorithm, even in complex problems. This is a significant advantage over other methods that can lead to suboptimal policies. The simplicity of the Q-Learning algorithm makes it a suitable candidate for use in this research. In contrast, the definition of the state space involves the creation of a vector comprising three components, designated ’states’. The first component signifies the vehicular demand of a particular antenna, the second component pertains to the allocation of resources of the antenna specified in the first component, and the third component represents the value of λ, which facilitates the verification of behaviour in terms of the available resources in the intelligent agent [39,50].

Based on the initial values, the reward calculation is carried out by evaluating the action applied to the current state. This process yields the next state, which is derived by considering both the penalties and rewards computed according to Equation (7).

(7) $$\mathrm{Rwrd}=rw\left(\mathrm{state}(i)-\mathrm{asig}(i)\right)+\mathrm{penalty}-\frac{\Lambda^{2}}{2}$$

The algorithm penalizes based on the results of applying these actions to the current states and thus producing new states. When the demand is greater than the allocation, the penalty is thus performed with a quadratic model of the form $-p^{2}$, where p>0. Conversely, when the demand is less than the allocation, the penalty is performed with a linear model of the form *p*, where (i.e., p<0). That is to say, the order of magnitude of $\left[-C,-C^{2}\right]$, including the value of 0.

The ensuing graphical representation offers a concise illustration of the resolution procedures, as depicted in Figure 3.

Finally, once the states, actions, penalties, rewards, and additional parameters are formally defined, the Q-value is computed. This value updates the Q-matrix by accumulating the expected rewards, to maximize the cumulative return over each episode and at time step $t_{i}$. Through multiple iterations, the algorithm progressively converges, leading to the reinforcement of an optimal learning policy for timely and efficient resource allocation. Upon completion of the learning process, the maximum Q values corresponding to each row of the Q matrix, each associated with a specific antenna state, are identified. This allows for the selection of the optimal action in each scenario, as defined by the SGNC. Over time, these selections consolidate into a stable decision-making policy that governs the agent’s behavior, ensuring optimal resource management.

### 3.3. Novelty and Contributions

This paper introduces a scalable and integrated framework for V2I infrastructure planning and radio resource management in dynamic urban environments, addressing key limitations of existing vehicular network studies that typically treat mobility modeling, infrastructure deployment, and resource allocation as independent problems.

The main novel contributions of this work are summarized as follows:

1. Joint integration of macroscopic mobility modeling and infrastructure planning: Unlike prior works based on static or microscopic assumptions, the proposed framework derives spatiotemporal V2I demand from realistic, georeferenced urban mobility patterns, enabling infrastructure decisions that adapt directly to congestion dynamics.
2. A dynamic ILP-based RSU deployment model with explicit coverage guarantees: An integer linear programming formulation is proposed to dynamically activate optimal subsets of RSUs from a large candidate set, achieving more than 90% vehicular coverage while activating less than 3% of the available infrastructure, thus significantly reducing deployment costs.
3. A generic reinforcement-learning-based controller with explicit saturation awareness: The Smart Generic Network Controller (SGNC) introduces a compact state representation and a λ-based saturation mechanism that enables stable and efficient radio resource allocation under high-demand conditions, without relying on deep or multi-agent learning architectures.
4. Scalability and stability under high load conditions: The proposed system maintains stable allocation behavior when the network exceeds 70% of its total capacity, dynamically reallocating resources in response to demand fluctuations, a scenario rarely addressed explicitly in existing RL-based V2I studies.

Overall, this work advances the state of the art by providing a unified, scalable, and demand-aware solution that bridges realistic mobility modeling, mathematical optimization, and reinforcement learning for next-generation intelligent transportation systems.

### 3.4. System Model and Environment Description

The proposed system is designed to operate in a dynamic urban V2I environment, where vehicular demand, infrastructure availability, and radio resources evolve over time. To clearly define this environment, the system model is structured into three interconnected layers that interact sequentially and continuously.

First, the *mobility layer* represents the urban traffic environment. Realistic, georeferenced road networks are extracted from OpenStreetMap and simulated using SUMO to generate macroscopic vehicular mobility patterns. This layer provides spatiotemporal information about vehicle density, positions, and traffic flow, which directly determines the demand for V2I communications at each time interval.

Second, the *infrastructure planning layer* uses the mobility information to dynamically select active Road-Side Units (RSUs). A set of candidate RSU locations is predefined over the planning area, and an Integer Linear Programming (ILP) model is applied at each time interval to activate the minimum number of RSUs required to satisfy coverage and capacity constraints. This layer ensures that a target percentage of vehicles is covered while minimizing infrastructure usage.

Finally, the *resource management layer* is implemented through the Smart Generic Network Controller (SGNC). The SGNC observes the system state, defined by vehicular demand, allocated radio resources, and the excess resource level (λ), and interacts with the active RSUs by dynamically reallocating resources using a Q-learning algorithm. This layer enables adaptive and stable resource management under fluctuating traffic demand and high-load conditions.

Overall, the environment is modeled as a closed-loop system in which mobility dynamics drive infrastructure activation, and infrastructure conditions guide intelligent resource allocation. This layered modeling approach improves clarity, scalability, and reproducibility, while accurately reflecting the behavior of real-world urban V2I networks.

#### Definition of Radio Resources

In the proposed system model, the term *radio resource* refers to an abstract and normalized allocation unit managed by an RSU to support V2I communications. Each radio resource represents a portion of the available transmission capacity and encompasses scheduling opportunities in the time–frequency domain, such as bandwidth slices or transmission slots, without binding the model to a specific physical-layer technology.

This abstraction enables the SGNC to dynamically allocate and withdraw resources based on vehicular demand and infrastructure load, while remaining independent of underlying standards (e.g., IEEE 802.11p or LTE-V2X) [20]. As a result, the model focuses on the efficiency and stability of resource management rather than on low-level radio configurations.

## 4. Results Analysis

**Figure 4:**
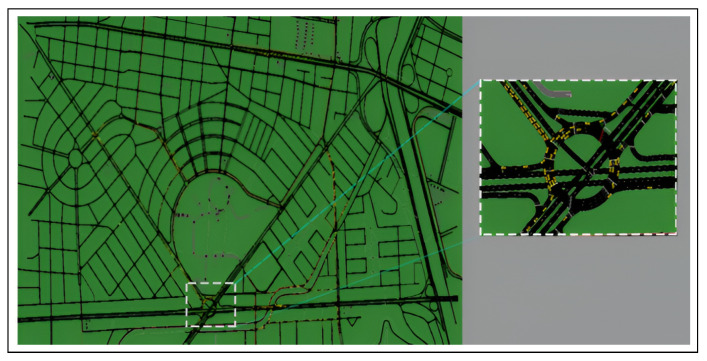
Mobility planning map.

Simulated mobility scenarios were generated based on realistic maps to assess user spatiotemporal demand and estimate traffic congestion levels. These maps incorporate vehicular flow and trace management, allowing for a rigorous analysis of traffic behavior and its potential impact on congestion. Figure 4 presents the planning map that depicts the study area’s street profiles, including key details such as loaded intersection points. This information is essential for constructing the initial mobility scenario and generating vehicle traces using an algorithm.

The generated trace, comprising 3,692,035 records, corresponds to the selected scenario. Each record contains variables such as: (i) time, (ii) the spatial coordinates of the vehicles, and (iii) unique labels that identify each unit vehicular within the simulation. This trace reflects the key dimensions of the simulated scenario, including the simulation’s total duration and the number of participating vehicles. Once the road topology is defined, vehicular flow is incorporated into the initial scenario.

The optimization process begins with the selection of a set of M=380 candidate RSU sites distributed throughout the planning region to ensure coverage across the entire map. The scenario is structured within a two-dimensional Cartesian coordinate system, where both axes represent rectangular coordinates measured in meters (m). This spatial configuration allows for precise localization and interpretation of the system’s components, facilitating an accurate assessment of resource distribution and vehicular demand across the coverage area. A total of *N* vehicles are progressively introduced into the simulated environment as the simulation time advances. At the end of the first interval, RSU activity is evaluated based on the model and the scenario illustrated.

The simulation results are obtained by instantiating the generic ILP formulation presented in Section 3 (Equations (1)–(6)) with the specific parameters of the considered scenario. In this example, the target coverage rate is set to ρ=0.9, and each RSU supports at most cap=12 vehicles. These values are used to build the ILP instances solved at each simulation interval. Therefore, no additional optimization formulation is introduced in this section, which focuses exclusively on the evaluation results.

Once the elements are installed on the stage and the equations are generated, they are processed using the integer linear programming model on the LPSolve package, which, when executed, presents optimization results as shown below.

**Figure 5:**
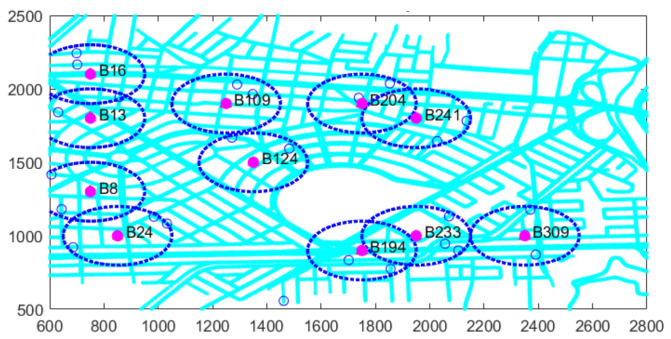
Example of a scenario with active candidate sites.

Table 5 shows that of the 380 candidate sites, only 11 were selected as optimal, representing 2.89% of the total. A total of 25 vehicles entered the planning area, of which 23 established connections with active RSUs, resulting in only 2 vehicles being out of coverage, corresponding to 8%. This result aligns with the 90% coverage guarantee criterion defined in the optimization model. This behavior is also visually confirmed in Figure 5.

The system gradually grows throughout the simulation, driven by the addition of more vehicles. This expansion also affects both the number and distribution of active candidate sites. The results obtained at each time interval reflect the deployment of the infrastructure, that is, the activation of candidate sites through a phase-by-phase optimization process. This dynamic provides a comprehensive view of the entire deployed infrastructure.

**Figure 6:**
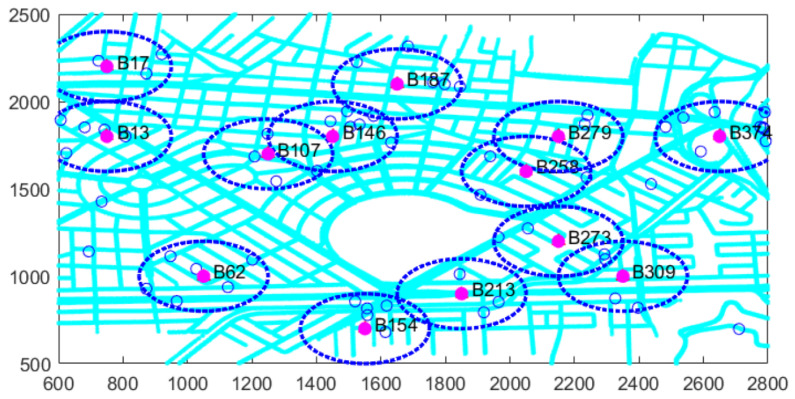
Example of a scenario with active candidate sites in second time intervals.

In contrast, in the subsequent dataset corresponding to the second time interval, a total of 62 vehicles entered the planning area, out of which 57 successfully established connections with active RSUs. Consequently, only 5 vehicles remained uncovered, representing 8.06%. Furthermore, only 13 candidate sites were selected as optimal, as illustrated in Table 6 and Figure 6.

**Figure 7:**
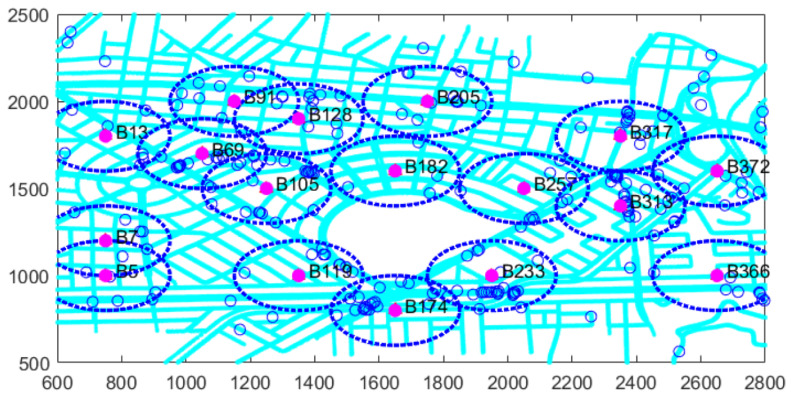
Example of a scenario with active candidate sites in ten time intervals.

The analysis of consecutive intervals yields different results in each case. Therefore, the example extends to the first ten intervals, as shown in Table 7 and Figure 7.

Specifically, in the final time interval presented, the results indicate that a total of 228 vehicles entered the planning area, of which 207 successfully established connections with active RSUs. Consequently, only 21 vehicles remained out of coverage, representing 9.21%, and merely 17 candidate sites were selected as optimal.

As shown in Table 7, it is possible to obtain the results corresponding to all previously analyzed intervals. These results provide valuable insights into the performance and behavior of the proposed model, with outputs consistently aligned with the predefined parameters. A key observation is that, as time progresses, the system’s complexity increases due to the continuous entry of additional elements, particularly a higher number of vehicles entering the scenario. Consequently, the set of active candidate sites tends to vary over time, reflecting the dynamic nature of the vehicular environment.

The analysis now continues with the resource allocation process, guided by the reinforcement learning algorithm proposed in the present study for the SGNC learning. To initiate the reinforcement learning algorithm, a set of numerical values is first defined: Nr=10, C=12, and PORC=70, corresponding to ten RSU antennas within the planning area, each with a maximum support capacity of twelve vehicles. The value 70 represents the percentage threshold of SGNC resources at which the resource allocation control analysis begins, after computing the variable CAP as follows: The value of the variable CAP is calculated as CAP=(Nr×C)×(PORC/100)=(10×12)×0.7=84. This value represents the resource usage threshold at which the SGNC enters an alert state to initiate a fair resource allocation process among the RSU antennas. Subsequently, the remaining capacity is calculated as: $LC=(N_{r}\times C)-\mathrm{CAP}=120-84=36$.

Table 8 illustrates that when the SGNC detects the utilization of 84 out of the 120 available resources, it initiates a detailed analysis process. With the increase of the setpoint, the SGNC progressively exhausts its available resources, prompting decisions to halt further allocations or, in critical scenarios, to revoke previously assigned resources. Despite operating under alert conditions, the system remains within its initial threshold, maintaining the capability to allocate RSUs according to current requirements.

Subsequently, the states are generated in $R^{2}$ through the construction of a two-dimensional table that contains all possible states defined as (0–12, 0–12, 0–36) = (state,assignment,λ). The first two components can take values between 0 and 12, while the third ranges from 0 to 36. Therefore, in this particular case, the problem size corresponds to a state space of order 13×13×37, that is, a matrix of size 6253×3 is obtained, containing all possible states in which the system state *S* can be found. Consequently, the states $S_{a}$ (current state) and $S_{n}$ (next state) can take values according to the order defined by the matrix, along with their corresponding indices (indest).

Additionally, the computation of the simulation visits to each state is performed. Then, the actions take values of −1, 0, or 1 depending on the case, and the Q-learning matrix is initialized to zero with dimensions 6253×3. Furthermore, other parameters are initialized, such as the execution time, which for the case study is set to the value num=200,000, γ=0.7, α=0.7, β=0.5, $\beta_{n}=0.01$. With these values, β tends to zero in each iteration.

Given the initial values from the first stage, the main loop execution is performed, consisting of *num* iterations of the learning process, covering all episodes (100 in this case) and all $N_{r}$ RSUs. Several calculations are carried out, including the random generation of states (*state*), resource allocation (*asig*), and the value of λ. Based on these values, the corresponding reward values Rwrd and Q-values are computed accordingly.

For greater clarity, examples are provided using the values obtained during the initial iterations of the main loop. Initially, the vector representing the current state of the RSU antennas is obtained. This corresponds to the vehicular demand at each RSU, which defines the overall system demand. In the first iteration, the following result was obtained, as shown in Table 9. Iteration 1:

The following values are then computed: the current state $S_{a}$ of antenna 1, denoted as A1; the resources assigned to it from the SGNC, denoted as asig(1); and the value of λ, calculated as described in the algorithm. Based on these, the following result is obtained, as shown in Table 10:

From the interpretation of the previous table, it can be observed that antenna 1 has a demand value equal to 9, and the SGNC assigns 9 resources accordingly. The corresponding λ value is 26, indicating that the SGNC can still allocate additional resources, although it has entered an alert state due to resource depletion. In this example, the demand exactly matches the number of resources assigned. The action and the penalty associated with this state are 0 and 0, respectively, as shown in Table 11.

At this point, the next state of the system ($S_{n}$) can be computed based on the action applied to the current state ($S_{a}$), as shown in Table 12.

With these values, the reward (Rwrd) is calculated as follows: Rwrd = rw(state(*i*) − asig(*i*)) + penalty −$(\lambda^{2})/2$; thus, Rwrd = rw(9 − 9) − 0 − (26^2^/2) = −338.

Finally, using the preceding values, the matrix Q is updated at the generated indices based on the corresponding state and action indices. Q= −235.9856, This value is calculated from Algorithm 2.

The objective is to calculate the *Q* matrix, which contains the system states in the rows and the possible actions in the columns. This matrix includes the rewards obtained, and the maximum value in each column indicates the action with the highest reward that should be applied to the corresponding state. The *Q* matrix reflects the system’s policies, which are the sum of the rewards derived from its best actions applied to each state, aimed at obtaining a favorable state. Table 13 illustrates the structure of the *Q* matrix together with the state matrix. Combining both matrices facilitates a comprehensive analysis of the problem’s dynamics by storing the rewards for each possible action in each state. The columns of the *Q* matrix represent actions such as withdrawing resources, modifying allocations, or allocating additional resources.

For example, for state (1002) = (2, 1, 2), where there are 2 users, 1 assigned resource, and 2 excess resources, the Q matrix displays the values (−1.8106, −1.7310, −1.6662). The maximum value of this vector is −1.6662, corresponding to the action of +1, which indicates that the SGNC must allocate additional resources to align with existing demand.

**Figure 8:**
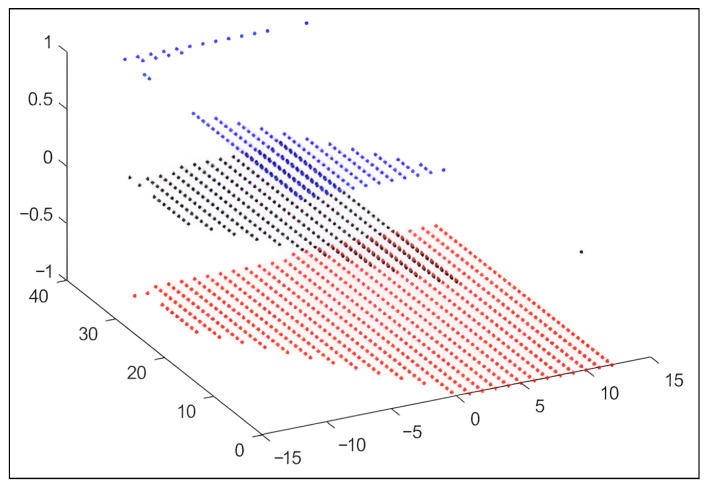
Relationship between demand and resource allocation, excess resources, and actions taken.

Figure 8 illustrates the relationship between demand, resource allocation, excess resources, and actions taken, enabling a detailed and accurate interpretation of the results. The figure’s x-axis represents the difference between demand and available resources, ranging from −15 to 15. Analysis of this axis shows that if the value is negative, demand exceeds the resources available in the RSU. If the value is positive, it indicates that the available resources exceed demand. A value of 0 reflects a balance between demand and resources, which is visualized in the graph by points located on the x-axis.

In Figure 8, the Y-axis represents the excess resources used. The behavior of these values varies according to the actions taken, which are shown on the Z-axis. The actions are classified into three categories: −1 indicates the action of withdrawing resources, 0 means no changes are made to the allocation, and +1 corresponds to allocating additional resources. When analyzing the relationship between these axes, it is observed that, as demand exceeds the allocated resources, the values tend to shift towards the negative region of the X-axis. Conversely, when resources exceed demand, the values change towards the positive region of the X-axis. In situations where demand and resources are close to each other (values close to zero on the X-axis) and excess resources are low (low values on the Y-axis), the SGNC policy favors allocating additional resources, represented by the action +1. On the other hand, when excess resource levels are high, the system chooses to remove resources from RSUs using action −1, thus optimizing resource utilization.

Generally, as allocated resources exceed demand and excess resource levels increase, the system adjusts its behavior by releasing resources to the SGNC. This dynamic enables efficient resource management, balancing allocation based on demand and detected excess resources, and ensuring the optimal use of available resources. The proposed approach places special emphasis on assessing the use of excess resources, as this factor is crucial in adjusting system policies.

**Figure 9:**
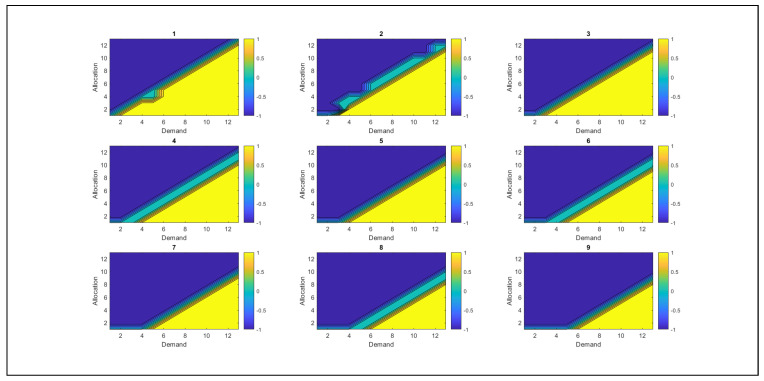
Graphical representation of optimal resource allocation policies.

As shown in Figure 9, the behavior of resources is presented, along with the derivation of optimal policies, which are the system’s best actions to achieve more favorable states. Figure 9 shows the relationship between demand and resource allocation, where colors identify them. The blue color represents action −1, corresponding to the withdrawal of RSU resources, whereas the cyan color indicates action 0, which implies that no changes have been made to the resource allocation. Yellow indicates action +1, which suggests allocating additional resources to the RSU. At this point, the value of λ triggers an alert in the SGNC, as the system has reached a level where resources begin to deplete.

The analysis reveals that, as overutilized resources increase, the SGNC controller tends to reduce resource allocation to RSUs. However, when demand is high and the allocated resources are insufficient to cover it, the SGNC continues to allocate additional resources because the excess resource levels are not yet significant. When the value of the excess resources increases significantly, the system adjusts its strategy and withdraws resources instead of allocating them. This behavior demonstrates the controller’s ability to adapt dynamically, balancing resource allocation according to demand and detected excesses, thereby avoiding overallocation and ensuring efficient management.

**Figure 10:**
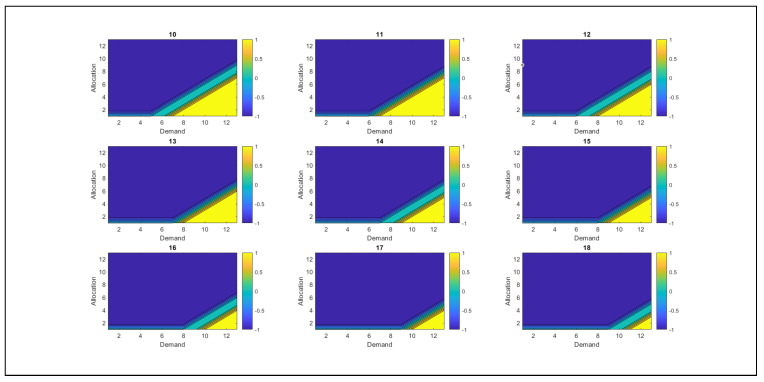
SGNC tendency to reduce resource allocation as excess resource use increases.

Figure 10 shows the system’s behavior as excess resource usage increases. As the value of the excess resources used grows, the SGNC progressively reduces resource allocation. This behavior is evident in the decrease in the yellow color (action +1, which allocates resources) and the increase in the blue color (action −1, which indicates the withdrawal of resources). When the excess resource usage reaches 50% of its maximum capacity (equivalent to 18 units in the case study), the resource allocation approaches zero. At this point, the predominant policy of the SGNC is to withdraw resources from the RSUs, reflecting an adjustment strategy aimed at avoiding over-allocation under conditions of excess. At this point, the value of λ is close to 50%, which allows the SGNC to be alerted that it has reached a medium alert level.

**Figure 11:**
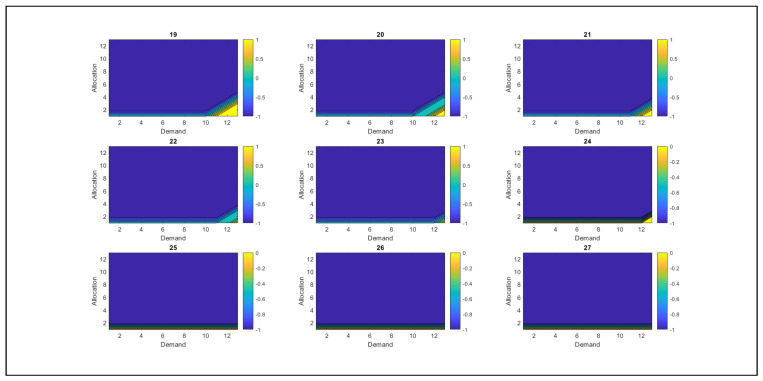
SGNC chooses to withdraw resources from the antennas, thus implementing the action policy −1.

Figure 11, corresponding to the remaining 50% of resources, shows behavior consistent with the previously described trend. Resource allocation is minimal: the yellow color, which represents positive allocation, appears only in small proportions, while the blue color, associated with resource withdrawal (action −1), predominates. This indicates that the SGNC has opted chiefly to withdraw resources from RSUs, which is logical considering that the resources available in the SGNC have already been exhausted. These results support the robustness and coherence of the proposed model, demonstrating that the solutions obtained adequately fit the conditions of the problem posed. At this point, the λ values are at their maximum, indicating that the SGNC has reached its highest alert level. This condition implies that resources must be withdrawn from the antennas, as they are nearly exhausted.

**Figure 12:**
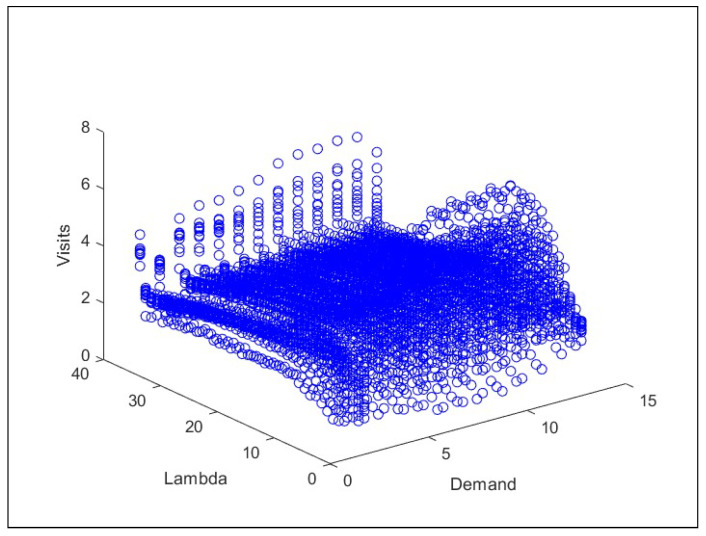
Relationship between demand, the value of Λ, and the number of visits to the states.

The analysis of visits to the states is presented in Figure 12. In this diagram, the x-axis represents the value of λ (excessively used resources), and the y-axis shows the total number of visits to each state. This diagram reveals that the highest frequency of visits occurs in states with λ values less than 50% of their maximum capacity. As λ increases, visits to these states decrease.

As can be seen in Figure 12, which shows the relationship between demand, the value of λ, and the number of state visits. It can be observed that the number of visits increases as demand grows from 0 to C. Subsequently, when λ reaches its maximum values between 0 and C/2, the number of visits begins to decrease at a rate of order $x\times 10^{6}$. To facilitate the interpretation of the results, a logarithmic function is applied that adjusts the scale of the data, providing a more precise representation of the trend.

In this study, the proposed algorithm addresses the intelligent allocation of resources through an SGNC agent, which learns from the environment, specifically from the dynamics of traffic flow influenced by vehicular demand within a V2I framework. This learning occurs at the RSU level, where a vector-based metric is defined for each RSU, comprising components such as the current demand, the assigned resources, and a dynamic lambda value (λ).

**Figure 13:**
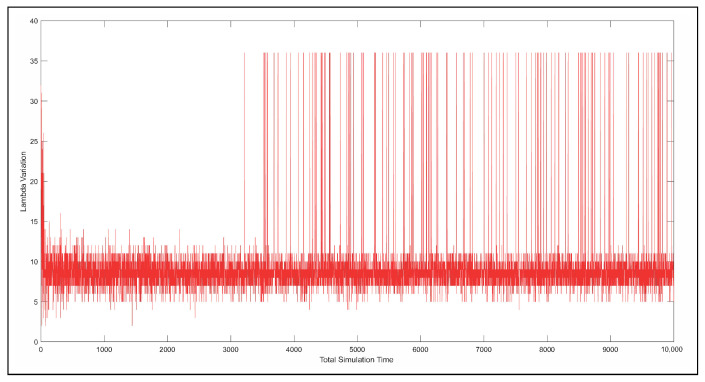
Relationship between demand, the value of Λ.

The λ parameter reflects the saturation level of each RSU in terms of resource utilization. When λ approaches its upper bound, it indicates that the SGNC is no longer capable of allocating additional resources to the RSU. Conversely, when λ remains within the lower or medium range, the agent retains the ability to allocate more resources to the RSU, thereby promoting system balance and operational efficiency. Figure 13 illustrates the variation of the λ parameter over simulation time.

In summary, a dynamic and macroscopic mobility scenario was initially obtained with respect to vehicular flow, which enabled an accurate estimation of traffic demand. Based on this foundation, the optimization model described in Algorithm 2 was applied, with its mathematical formulations defined in Equations (1)–(6). This model allows the selection of active candidate sites to ensure optimal coverage. The simulation results are presented in Table 5, Table 6 and Table 7, within the Results Analysis section, where the partial values for each time interval are included.

Table 5 shows the initial scenario with M=380 candidate sites. Based on the vehicular demand, the optimization model selected 11 active RSUs, representing only 2.9% of the total sites. For this first simulation, N=25 vehicles were considered, of which the model successfully covered 23, while 2 remained uncovered, corresponding to 8% of unmet demand.

It is important to note that vehicles dynamically enter and leave the planning area, and simulations were executed in intervals of 40 time units. Table 7 summarizes the results across 10 intervals, defined as follows: $t_{1}=40$ for the first interval, $40<t_{i}\leq 80$ for the second, $80<t_{i}\leq 120$ for the third, and so on.

Subsequently, the reinforcement learning model was trained through Algorithm 2, whose nomenclature is detailed in Table 3. In this process, the agent—defined as the SGNC—was configured with its respective states, actions, and rewards, which are essential elements for its operation. The training dynamics are exemplified in the Results Analysis section preceding Table 8, where the SGNC’s resource utilization and the alerts triggered for resource allocation to active antennas are described.

In a particular case, $N_{r}=10$ active antennas were considered, each capable of serving up to 12 vehicles. In this scenario, the SGNC manages a total of 120 resources for distribution. However, from resource number 85 onward, alerts were triggered, which activated the intelligent allocation of the remaining 36 resources through a λ variable. This mechanism enabled the SGNC to learn how to manage resources efficiently as they were depleted, avoiding ineffective assignments and freeing resources within the vehicular infrastructure.

The results of this process are provided in Table 9, Table 10, Table 11, Table 12 and Table 13, while Figure 8 illustrates the relationship among demand, resource allocation, surplus, and the actions taken in policy formulation. Finally, Figure 10 and Figure 11 demonstrate the stability achieved in the overall resource allocation. The results obtained validate the effectiveness of the proposed optimization model and the reinforcement learning scheme based on the SGNC. In the first phase, the selection of active candidate sites achieved coverage above 90% of the vehicles in the scenario, while using only 2.9% of the available infrastructure. This confirms the model’s ability to significantly reduce deployment costs without considerably compromising quality of service. In the second phase, the SGNC training showed stable performance in resource allocation, intelligently managing the limitations introduced by the λ variable. The agent was able to equitably distribute the available resources, preventing congestion and ensuring an adequate balance between demand and network capacity.

Finally, the analysis of Table 7 and Figure 8, Figure 10 and Figure 11 reveals that the combination of mathematical optimization and reinforcement learning not only enhances initial coverage but also provides resilience and stability in resource management under dynamic vehicular scenarios. These results confirm the relevance of the proposed approach for future implementations in intelligent vehicular communication systems.

This work introduces an optimization model for dynamic vehicular mobility scenarios that efficiently selects active candidate sites based on traffic demand, achieving coverage levels above 90% while utilizing only 2.9% of the available infrastructure. A reinforcement learning agent (SGNC) is further integrated, configured with specific states, actions, and rewards, enabling adaptive and intelligent resource allocation under varying conditions. A key contribution lies in the efficient resource management mechanism through the λ variable, which ensures balanced distribution even in high-demand scenarios, thereby maintaining stability in the allocation process. Collectively, these contributions establish a methodological framework that combines mathematical optimization and reinforcement learning to enhance coverage, reduce deployment costs, and improve the resilience of resource management in intelligent vehicular networks.

### Comparison with Existing Dynamic Resource Allocation Approaches

Most existing dynamic resource allocation strategies in vehicular networks rely on heuristic or threshold-based mechanisms that adjust resources according to instantaneous load or predefined congestion indicators. While these approaches improve upon static allocation, they often lack long-term stability and do not explicitly account for system-wide resource saturation.

Recent studies have also explored reinforcement-learning-based solutions, including deep and multi-agent variants, to dynamically allocate V2I resources. Although effective in adaptive scenarios, many of these approaches assume either abundant resources or semi-static conditions, and rarely analyze system behavior near or beyond capacity limits.

In contrast, the proposed SGNC introduces an explicit saturation-aware mechanism through the λ parameter, which continuously reflects the level of excess resource usage at the infrastructure level. This design enables the controller to proactively regulate allocations, preventing over-provisioning and maintaining stable operation even when resource utilization exceeds 70% of total capacity.

Compared with heuristic dynamic schemes, the SGNC demonstrates improved stability and fairness in resource redistribution. Compared with existing RL-based methods, it achieves adaptive behavior using a compact state representation and without requiring deep neural networks or multi-agent coordination, significantly reducing computational complexity while preserving performance.

This comparison highlights that the main advantage of the proposed approach lies not only in adaptability, but also in robust and scalable operation under high-load conditions, which are common in dense urban V2I environments.

## 5. Conclusions

This paper presents a dynamic model for vehicular network resource allocation (SGNC) that adapts to the variability of traffic flow and resource demand. The results show that the SGNC adjusts its allocation policy as excess resource usage increases. It does so by progressively reducing the resources allocated to RSUs and eventually removing them when a critical threshold is reached. This progressive reduction is a key feature of the model, ensuring efficient management, optimizing resource allocation, and preventing overallocation, thereby improving the system’s responsiveness under high-demand conditions.

Based on reinforcement learning, the model developed for simulating vehicular traffic behavior and dynamic resource allocation proved effective in adapting to changes in mobility and network topology. It incorporated optimal policies and controlled excess resources using the λ parameter, allowing for more informed allocation decisions and thereby improving overall system efficiency. These findings provide a solid foundation for future research and advancements in vehicular network optimization.

Despite the progress made, several areas remain for future research and improvement. The potential of implementing advanced optimization techniques, such as deep learning, is promising. Furthermore, it is suggested that the model be expanded to include additional factors, such as vehicle mobility and network conditions, to evaluate its performance in larger-scale networks and more complex scenarios. Methods for differential resource allocation based on service types could also be investigated. However, validating the model in real-world environments is crucial to ensure its effectiveness under practical conditions. These lines of work will improve the system’s efficiency and expand its applicability to more dynamic and realistic contexts.

The analysis of resource allocation metrics in vehicular networks, the primary objective is to optimize the utilization of spectrum, transmission power, and time. These parameters vary depending on the network architecture and the communication technologies employed. In this context, our study places special emphasis on vehicular networks with high traffic demand, particularly in urban roadways, where the operational dynamics significantly impact society due to the frequent incidents occurring in these areas and the consequent economic and social losses they generate.

## Figures and Tables

**Table 1:** Comparison of related work in intelligent vehicular networks.

Study/Approach	Technique Used	Differentiator Compared to the Proposed Work
SUMO/OSM and traffic simulators	Microscopic and macroscopic models, VANETs using real maps	Integration of realistic mobility with adaptive and scalable intelligent agents in real time
RSU placement optimization (evolutionary algorithms, LP)	Multi-objective algorithms on fixed V2I infrastructure	Distributed and scalable scheme based on vehicle density and demand
Statistical inference and ML for congestion detection	Supervised ML prediction on V2V/V2I networks	Combines prediction with autonomous action through intelligent agents
IEEE 802.11p/LTE-V2X protocols	Vehicular communication standards for ITSs	Spectrum adaptation using mobile intelligent nodes
RL and deep learning for resource allocation	DQN, MARL, DDPG in semi-static scenarios	Mobile distributed agents learning in real-time dynamic environments
Game theory models (congestion, Nash)	Non-cooperative games, semi-Markov models on V2I/RSUs	Distributed optimization with lower complexity and adaptive reconfiguration
Our work	Distributed and adaptive RL-based intelligent agent on mobile infrastructure (UAVs/vehicles)	First to integrate mobility, on-demand scalability, and real-time distributed learning

**Table 2:** Vehicle positions over time.

Time	X-Axis	Y-Axis	Vehicle Label
$t_{0}$	$x_{0}$	$y_{0}$	$e_{0}$
$t_{1}$	$x_{1}$	$y_{1}$	$e_{1}$
$t_{2}$	$x_{2}$	$y_{2}$	$e_{0}$
$t_{3}$	$x_{3}$	$y_{3}$	$e_{2}$
⋮	⋮	⋮	⋮
$t_{n}$	$x_{n}$	$y_{n}$	$e_{n}$

**Table 3:** Key symbols used in the SGNC learning model.

Symbol	Domain	Definition
**Variables**		
Nr	$Z^{+}$	Number of antennas in the system
*C*	$Z^{+}$	Capacity per antenna
PORC	∈{0,100}	Assignment guarantee rate
CAP	$Z^{+}$	SGNC resource capacity
LC	$Z^{+}$	Max value for Λ
num	$Z^{+}$	Total execution time
α	∈(0,1)	Learning rate
β	→0	Exploration suppression
γ	∈(0,1)	Discount factor
*K*	$R^{+}$	Beta distribution parameter
time	∈{1,…,num}	Current simulation time
Λ	∈(0,LC)	Excess resource capacity
episodes	$Z^{+}$	Training iterations
*a*	∈{−1,0,1}	Selected action
Rwrd	∈R	Reward for state–action pair
iSa	∈{1,…,|S|}	Index of current state
iSn	∈{1,…,|S|}	Index of next state
**Arrays**		
States	$\in R^{2}$	Set of all RSU states
Indest	∈R	State index mapping
tabla4	$\in R^{2}$	MDP transition matrix
actions	∈{−1,0,1}	Action vector
*Q*	$\in R^{2}$	Policy matrix
state	∈R	Demand vector
asig	∈R	Resource allocation vector
Sa	∈{C,C,LC}	Current state at time *t*
Sn	∈{C,C,LC}	Next state after action
visits	∈R	Visit count per state

**Table 4:** List of symbols, sets, parameters, and decision variables.

Symbol	Domain	Description
**Sets**		
*Y*	$\in \{y_{1},y_{2},\ldots ,y_{N}\}$	Set of vehicles
*Z*	$\in \{z_{1},z_{2},\ldots ,z_{M}\}$	Set of candidate site
*E*	$\in \{e_{1},e_{2},\ldots ,e_{N}\}$	Vehicle identification labels
$I_{t}$	$\in \{t_{1},t_{2},t_{3},\ldots \}$	Set of time intervals
**Parameters**		
*M*	$Z^{+}$	Number of candidate sites
*N*	$Z^{+}$	Number of vehicles
ρ	$R^{+}$	Coverage percentage
cap	$Z^{+}$	Antenna capacity
*R*	$Z^{+}$	Antenna range
num_interv	$Z^{+}$	Number of time intervals
**Decision variables**		
$z_{i}$	∈Z	*i*-th RSU site
$y_{j}$	∈Y	*j*-th vehicle
$X_{i,j}$	$\in \{X_{1,1},X_{1,2},\ldots ,X_{M,N}\}$	Connectivity variable
$\alpha_{i,j}$	{0,1}	Binary connectivity decision

**Table 5:** Example of optimization results for the first interval.

M	Act. RSUs	% RSU	N	Veh. Cov.	Veh. Uncov.	% Uncov.
380	11	2.9	25	23	2	8.0

**Table 6:** Example of optimization results for the first and second time intervals.

#	M	RSU Opt.	% RSU	N	Veh. Cov.	Veh. Uncov.	% Uncov.
1	380	11	2.8947	25	23	2	8.0000
2	380	13	3.4211	62	57	5	8.0645

**Table 7:** Example of optimization results for the first ten time intervals.

#	M	RSU Opt.	% RSU	N	Veh. Cov.	Veh. Uncov.	% Uncov.
1	380	11	2.8947	25	23	2	8.0000
2	380	13	3.4211	62	57	5	8.0645
3	380	15	3.9474	97	89	8	8.2474
4	380	18	4.4737	123	111	12	9.7561
5	380	17	4.4737	131	115	16	9.6870
6	380	17	4.4737	145	129	16	9.6793
7	380	18	4.7368	172	155	17	9.8837
8	380	18	4.7368	209	190	19	9.0904
9	380	18	4.7368	218	199	19	8.7156
10	380	17	4.4737	228	207	21	9.2105

**Table 8:** Example of SGNC resource usage and associated alerts.

SGNC Resource Usage	Setpoint ϵ LC	Alerts
85	1	Warning 1
86	2	Warning 2
119	35	Warning 35
120	36	Warning 36

**Table 9:** Example of antenna demand (state vector).

A1	A2	A3	A4	A5	A6	A7	A8	A9	A10
9	7	0	4	2	7	11	0	11	11

**Table 10:** Example of current state.

Current State *i* ($S_{a}$)	Asig *i*	λ
9	9	26

**Table 11:** Action and penalty associated with the current state.

Action	Penalty
0	0

**Table 12:** Computed values after applying the action to the current state.

Next State *i* ($S_{n}$)	Asig *i*	λ
9	9	26

**Table 13:** Structure of the *Q* matrix associated with different states and actions.

State	−1m	0m	+1m
(2, 1, 2)	−1.8106	−1.7310	**−1.6662**

## Data Availability

Data supporting the reported results can be obtained from the corresponding author upon reasonable request.

## Abbreviations

The following abbreviations are used in this manuscript:

ITSs	Intelligent Transportation Systems
VANET	Vehicular Ad Hoc Network
V2I	Vehicle-to-Infrastructure
V2V	Vehicle-to-Vehicle
RSU	Road-Side Unit
SGNC	Smart Generic Network Controller
RL	Reinforcement Learning
ML	Machine Learning
LTE-V2X	Long-Term Evolution Vehicle-to-Everything
OSM	OpenStreetMap
SUMO	Simulation of Urban Mobility
MDP	Markov Decision Process
Q-Learning	Q-Learning Reinforcement Algorithm
DQN	Deep Q-Network
RGB	Resource–Gain Balance (from reward formulation)
CAP	Resource Capacity Threshold
LC	Excess Resource Capacity
PORC	Resource Assignment Percentage
